# Public Health Impact of the MVA-BN Vaccine During the 2022 Mpox Outbreak: A Systematic Review

**DOI:** 10.3390/idr17050124

**Published:** 2025-10-07

**Authors:** Sarah C. Katsandres, Suzanne K. Scheele, Takako Kiener, Lisa Bloudek

**Affiliations:** 1 Curta, Inc., Seattle, WA 98104, USA; sarah.katsandres@curta.com (S.C.K.); takako.kiener@curta.com (T.K.); lisa.bloudek@curta.com (L.B.); 2 Bavarian Nordic Inc., Durham, NC 27703, USA

**Keywords:** mpox, monkeypox, MVA-BN, mpox vaccine, vaccination, cases averted, epidemics, public health, vaccine impact, outbreak prevention

## Abstract

Background/Objectives: Previously endemic to sub-Saharan Africa, mpox has since emerged globally, resulting in more than 150,000 cases in over 100 countries in the 2022 outbreak. The Modified Vaccinia Ankara-Bavarian Nordic (MVA-BN) vaccine is licensed and recommended for at-risk populations in many countries and received World Health Organization (WHO) pre-qualification in September 2024. Methods: We conducted this systematic literature review (SLR) to compare analyses, published from 2022 through 2024, of cases averted due to mpox vaccination during the 2022 outbreak to assess the feasibility of estimating the impact on the United States (US). The search included studies that utilized reported case data from any country. Results: Nine studies were identified. Four estimated the impact of the vaccine as directly modeled in the 2022 outbreak, and cases averted ranged from 10% to 79%. One assessed the projected impact on future outbreaks. Four estimated the impact of hypothetical vaccination strategies. Only one model utilized assumptions appropriate for the US outbreak and population, to allow for an estimate of US cases averted (53,499 cases averted due to the synergistic effects of the vaccine and behavioral changes, with 8096 due to the mpox vaccine alone and 5478 due to behavioral changes alone). Conclusions: Variation in estimates for the impact of the vaccine can typically be explained by differing model approaches, assumptions, inputs, and epidemic peaks and vaccination campaign roll-out. Most models were not generalizable to the US outbreak and population, but one yielded a reasonable estimate. Nevertheless, all models emphasized the importance of vaccination combined with other public health interventions.

## 1. Introduction

Mpox disease is a viral illness caused by the monkeypox virus (MPXV), of the genus *Orthopoxvirus*, spread through contact with infected animals or close skin-to-skin contact with an infected human [1]. Historically, mpox has been endemic in several African countries where surveillance data are limited, but cases have been thought to be on the rise in recent years due to the cessation of smallpox vaccination, which provided some cross-protection [2].

In 2022 a large global outbreak of mpox from clade IIb MPXV occurred, which was the first sustained mpox transmission outside of Africa since the first human case was identified in 1970. The outbreak was initially concentrated in Europe and the Americas before spreading to over a hundred countries. The World Health Organization (WHO) declared the outbreak a public health emergency of international concern (PHEIC) on 23 July 2022, and the United States (US) declared it a public health emergency on 4 August 2022 [3]. Based on data published on 10 January 2024 by the US Centers for Disease Control and Prevention (CDC), this global outbreak led to an estimated 99,500 cases and 200 deaths worldwide, of which 32,000 cases and 60 deaths occurred in the US [4]. Approximately 96% of reported cases occurred in locations that have not historically reported mpox prior to this outbreak. This global outbreak was associated primarily with sexual transmission. Gay and bisexual men who have sex with men (GBMSM) were the most affected, representing about 86% of cases globally as of July 2024 [5].

The Modified Vaccinia Ankara-Bavarian Nordic (MVA-BN) (JYNNEOS^®^, Bavarian Nordic A/S, Hellerup, Denmark) is a live-attenuated vaccine, approved by the Food and Drug Administration (FDA) in September 2019 (and by other countries), for the prevention of smallpox and mpox disease. The first reported doses given in response to the outbreak in the US were during the week of 22 May 2022 at just 50 doses, with the peak number of first dose administrations, of the two-dose recommended schedule, reaching 104,702 during the week of 7 August [6]. The peak number of second doses administered was not until the week of September 18th, which was at the epidemic’s peak (reaching 467 reported cases in a single day on 6 August 2022) [7].

MVA-BN is currently licensed and recommended for use in adults at risk of mpox infection in the US, European Union (EU), Canada, and the United Kingdom (UK), among other countries [8,9,10,11]. The vaccine has been deployed in several African countries under Emergency Use Authorizations and WHO Emergency Use Listing (EUL) and pre-qualification following the dramatic upsurge in mpox cases in the Democratic Republic of Congo (DRC) in 2024 [12]. On 14 August 2024, the WHO declared the situation in the DRC and the outbreak’s spread to several other African countries to be a PHEIC. Hundreds of thousands of doses have been delivered to affected sub-Saharan countries, some of which have since implemented vaccination campaigns [13,14].

Multiple studies have attempted to measure the incremental impact and synergistic public health impact of the mpox vaccine on the 2022 outbreak and on future outbreaks in different geographic locations and using different modeling approaches. Taken together, these studies help to characterize the impact of vaccination strategies in the context of concurrent behavioral interventions to determine needs for future outbreaks and vaccination campaign planning. However, these models have focused primarily on North American and European populations and GBMSM sexual transmission dynamics that dominated the 2022 outbreak. Therefore, these results cannot necessarily be extrapolated to the ongoing outbreak in the DRC and elsewhere in Africa.

## 2. Materials and Methods

The objective of this systematic literature review (SLR) was to identify all studies which evaluate the public health impact of the mpox vaccine during the 2022 outbreak in terms of cases averted due to the vaccine. The vaccine’s impact is examined in a variety of settings and in the context of public health interventions such as promotion of behavioral change, in order to estimate the number of US cases that were averted during the 2022 outbreak.

This SLR followed the Preferred Reporting Items for Systematic Reviews and Meta-Analyses (PRISMA) guidelines [15]. The search was conducted in MEDLINE (via PubMed) for peer-reviewed articles published in English from 1 January 2022 through 10 January 2024. Modeling studies which sought to measure the real-world case data against a counterfactual, modeled scenario in which no vaccine existed during the outbreak and reported the number or proportion of cases averted due to the mpox vaccine were included. There were no restrictions on population (age, gender, or ethnicity) or geographical setting. Studies were excluded that estimated public health impact due to behavioral change alone, but if a study estimated impact of the vaccine in combination with behavioral change or other control measures, it was included. Studies that modeled purely hypothetical scenarios (e.g., to simulate vaccination distribution scenarios or to assess varying vaccine efficacy) but did not use actual case data or trends were excluded. All citations were screened first by title and abstract and then by full text and were reviewed by two screeners each using the Nested Knowledge literature review platform (Version 1.78.1, Nested Knowledge, St. Paul, MN. USA) [16]. Any conflicts were resolved by a third reviewer. Three authors (S.C.K., T.K., and L.B.) screened citations at both levels and adjudicated conflicts, as needed. The primary outcome included cases averted due to the vaccine (number or proportion), or due to the vaccine and other control measures, and predicted future cases averted due to the vaccine. The full search strategy can be found in Supplemental Table S1. The protocol was not pre-registered in a public registry.

Studies were assessed for the feasibility of using the findings to quantify the number of US cases that were averted during the 2022 outbreak due to the mpox vaccine. If determined that model parameters and assumptions were deemed appropriate for the US, the proportion of cases hypothetically averted due to the vaccine was applied to the actual daily US mpox case numbers published by the CDC for the 2022 outbreak after vaccine introduction, in order to calculate the number of cases on national scale.

## 3. Results

### 3.1. SLR Findings

**Figure 1:**
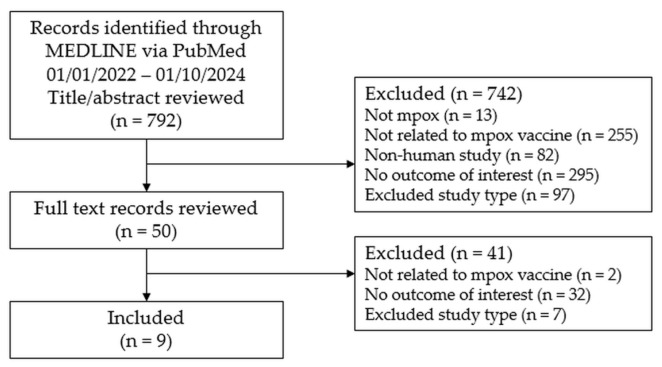
PRISMA Diagram.

A total of 792 records were screened and 50 underwent full text review. In total, nine full text articles met all inclusion and exclusion criteria. Figure 1 shows the full PRISMA diagram.

This SLR identified four publications that estimated the impact of the mpox vaccine on the 2022 outbreak, one that estimated the impact of the mpox vaccine on a future outbreak, and four that estimated the impact of hypothetical vaccination programs or strategies on the 2022 outbreak (Table 1). The included studies, grouped by the impact they aimed to quantify in Section 3.2, Section 3.3 and Section 3.4, are discussed individually due to the heterogeneity of model structure and objectives. A comparison of the studies’ methods, results, key parameters, and geographical settings is presented in Table 1.

### 3.2. Studies That Measured the Impact of the Vaccine During the 2022 Outbreak

#### 3.2.1. Lin, 2024 [17]

Lin et al. estimated the impact of the vaccination campaign and behavior changes in the US. They used a deterministic, risk-structured model and US data between 22 May and 22 December 2022. The basic reproduction number (R_0_) used in their model was 3.88 in the high-risk group and 0.39 in the low-risk group. It was estimated that 71.8% of mpox cases originated from the high-risk group. The model estimated that a two-dose vaccination campaign alone could have prevented 21.2% (95% confidence interval (CI): 10.2–24.1%) of cases, and that behavior changes alone could have prevented 15.4% (14.3–20.6%) of cases. The combination of both measures had a synergistic effect and was likely to have prevented 64% (95% CI: 43.8–60.9%) of cases in total. The model made the following key assumptions: a ten-fold difference in transmission potential between the high-risk and low-risk group and a reduction in behavior at-risk for transmission in the high-risk group that would be maintained until at least the end of 2022.

#### 3.2.2. Zhang X, 2024 [18]

Zhang et al. assessed the impact of control measures on the 2022 outbreak among the GBMSM population in England. They sought to understand the reasons for the outbreak’s downturn and used two deterministic compartment models: a structured model in which the GBMSM population was stratified by transmission risk level (low vs. high) and a homogenous model in which everyone had the same transmission risk. The structured model used an R_0_ of 1.94 in the high-risk group and 0.67 in the low-risk group, and the homogenous model used multiple methods to estimate R_0_, which ranged from 1.41–2.17. The model estimated that vaccination alone (if no behavioral changes occurred) would have prevented 9.8% (16,441 cases) of infections in England and minimized a resurgence of cases in January 2023. The study also showed that an earlier initiation of the vaccination campaign could have prevented 4.5 times more infections. Vaccination was estimated to have only marginally increased the number of cases averted, and when added to the scenario with the other interventions, prevented an additional 0.1% of cases. Additionally, targeting vaccines to high-risk groups first could achieve herd immunity thresholds at a vaccination rate of only 5%, in contrast with simple estimates of between 23.6–29.6% based on the R_0_ estimates. The model assumed 50% of the high-risk group gradually reduced behavior at-risk for transmission starting 22 May, due to an awareness of the outbreak and the risk for transmission.

#### 3.2.3. Brand, 2023 [19]

Brand et al. investigated factors leading to the decline in mpox incidence in the UK. The results of a stochastic, discrete population transmission model using UK case data suggested that the decline in mpox cases was primarily due to changes in behavior at-risk for transmission and immunity from previous infections. While vaccination did not begin in London until the 16th of July, the combined effects of natural infections and vaccinations were estimated to have reduced the effective reproductive number by 74.7% (95% prediction interval (PI): 59.6–94.1%) by the first week of September 2022. The diminishing risk of transmission, however, was likely to be due to behavioral change and population immunity. In addition, vaccination likely helped prevent a resurgence of cases by being concentrated among the GBMSM population. In the study, the GBMSM population was subcategorized based on their sexual activity and thus had varying probabilities of the number of partners infected with mpox. Subsequently, vaccine distribution was modeled as proportionate to the subgroup size.

#### 3.2.4. Clay, 2024 [20]

Clay et al. evaluated the impact of vaccination and behavioral changes on the 2022 mpox outbreak in Washington DC, US. A dynamic network transmission model using mpox case data found that initial declines were due to alterations in behavioral at-risk for transmission. After one year, there was an estimated 84% reduction in cases (interquartile range (IQR) 67% to 91%) due to the combination of vaccination and behavioral change. From vaccination alone, 79% (IQR 64% to 88%) of cases were prevented, and 25% (IQR 10% to 42%) of cases from behavioral adaptations alone. The results suggest that the outbreak would have not ended without vaccination and that behavioral changes alone may have reduced cases but ultimately prolonged the outbreak due to a resulting shift in the outbreak’s peak. Of note, the study used the level of Reddit activity among LGBTQ+ communities, within subreddits related to mpox, as a proxy for risk perception, which informed the model’s behavioral adaptation parameters, specifically the reduction in sexual activity at-risk for transmission.

### 3.3. Studies That Measured the Impact of the Vaccine on a Future Outbreak

#### Shamier, 2024 [21]

Shamier et al. investigated the impact of immunity conferred by prior infections and vaccination on a potential future mpox outbreak in the Netherlands. A stochastic transmission model using Netherlands serological data found that without the 2022 vaccination campaign a future outbreak would be only marginally smaller than the original: 1427 (IQR: 1321–1565) vs. 1325 (IQR: 1262–1419), respectively. The study also found that with vaccination and no changes to behavior, the future outbreak would be reduced to only 344 cases. Moreover, with a partially vaccinated population and a shorter time-to-diagnosis, it was predicted that no outbreak would occur. The study underscored the importance of rapid diagnostics in achieving comprehensive prevention of transmission. It was assumed the following: individuals ceased high-risk behavior and did not transmit the virus after diagnosis, there would be a reduction in sexual partners by up to 50% after 23 May due to increased awareness, and a hypothetical turnover rate of 1% to 5% per year was applied to the sexually active Dutch GBMSM population. These findings rely on the assumption that the smallpox vaccine results in cross-protective neutralizing antibodies against mpox virus in humans, which the authors state has been corroborated in recent research [26,27,28,29]. The researchers had previously demonstrated that individuals who were vaccinated for smallpox up to 70 years before the mpox outbreak of 2022 still had detectable neutralizing antibodies. Additionally, the longevity of the immune response elicited by the mpox vaccine must be great enough to prevent future outbreaks, for their estimates to stand. Recently however, there have been breakthrough infections in those previously vaccinated, bolstering the evidence for waning immunity and declining Immunoglobulin G antibody levels, seen predominantly among those who had not been vaccinated against smallpox as children, according to the authors [30,31]. Ultimately, they acknowledged that simulated outbreaks 10 years from now will likely underestimate the total case numbers, though the duration of protection from the vaccines remains unknown.

### 3.4. Studies That Measured the Impact of a Hypothetical Vaccination Program or Strategies

#### 3.4.1. Zheng, 2022 [22]

Zheng et al. assessed the effect of diagnostic interventions and ring vaccination on the transmission dynamics of the 2022 mpox outbreak in 44 states in the US. The study used an epidemic dynamic model and simulated interventions and vaccination strategies up until 15 July 2022. The model estimated that ring vaccination, covering 20% of exposed contacts, reduced mpox cases by 32% by the third quarter and 61% by the end of the year. Reducing the delay from disease onset to diagnosis from nine days to five or even three days was determined to reduce cumulative infections by 97% and 99%, respectively, by the end of 2022.

#### 3.4.2. Knight, 2022 [23]

Knight et al. modeled the optimal vaccine allocation strategy between two transmission networks to maximize the limited vaccine supply among GBMSM. Using a deterministic compartmental transmission model with 2022 data from two cities in Ontario, the study simulated vaccine distribution over 30 days. The results suggest that the optimal allocation strategy, which yielded the fewest infections by day 90, involved prioritizing larger networks with more initial infections or those with higher R_0_. Under conditions in which there are fewer initial cases but a higher R_0_, distributing doses between the two cities was optimal. The city specific R_0_ values were calculated assuming no interaction or mixing of populations between cities. Optimal allocation of the vaccination supply was strongly influenced by the relative R_0_ values between the cities, the share of cases that seeded the infection, and the size of the city.

#### 3.4.3. Gan, 2023 [24]

Gan et al. assessed the impact of different vaccination uptake levels to make recommendations for preemptive vaccination strategies for the GBMSM community in three major global locations: Singapore, Hong Kong, and Sydney. An individual-based compartmental model was used to assess three levels of vaccination uptake: 25%, 50%, and 80%. Model estimates suggest that mass vaccination over one year at these coverage levels could reduce total cases by 22–29%, 45–59%, and 71–96%, respectively. The average number of cases averted per targeted vaccine administration was estimated to range from 0.86 to 1.47, depending on the geographic location. The key assumptions of this study were that the size of the GBMSM population in all three locations was 3% of the city’s working population of men and that behavior at-risk for transmission remained unchanged, even after mpox infection. This last assumption is different from other models, which assume a reduction in activity to some level following infection. The model was parameterized using pre-coronavirus disease 2019 (COVID-19) sexual behavior surveys from the US, Hong Kong, and Sydney, which may not reflect recent trends.

#### 3.4.4. Zhang L, 2024 [25]

Zhang et al. simulated global mpox transmission, vaccination, and control scenarios using a modified susceptible, exposed, infected, recovered (SEIR) model. The results of the model suggested a 29% reduction in infectious cases when 30% of the population was vaccinated and a 16% reduction with 20% vaccination coverage. The US, Brazil, Spain, France, the UK, and Germany were six countries included in the study, with predicted case numbers of 30,543, 11,191, 7447, 5945, 5606, and 4291, respectively. The model assumed an all-or-nothing vaccine type, rather than the leaky vaccine type assumed by most other modeling studies, which assumes that only 78% of vaccinated individuals would be fully protected from mpox. It was also assumed that all vaccinations were completed prior to the outbreak’s onset. Additionally, the model did not account for other control measures that may have influenced case numbers, such as a reduction in interactions following awareness of the outbreak.

### 3.5. Estimate of Cases Averted in the US

One analysis provided the structure and parameters most appropriate to the US population: Lin et al., 2024. This study provided estimates of the proportion of cases avoided by vaccination alone, behavior alone, and synergistic combination of vaccine and behavior [17]. These proportions were used to calculate a counterfactual—the number of cases that would have been observed in the US in the absence of the vaccine or behavioral modification. The difference between this counterfactual and the actual number of cases observed in the US yields an estimate of cases averted due to interventions. Using this approach, 8096 cases were avoided due to the mpox vaccine alone. However, as many as 53,499 cases were avoided by the vaccine and its synergistic effect with behavioral changes and 5478 cases due to behavioral changes alone. In this model, the vaccine on its own had a larger impact on disease reduction than behavioral change alone. But the synergistic effect of the two combined was five-fold and eight-fold greater than either behavior change or vaccination alone, respectively.

The results of other studies were deemed unsuitable to extrapolate to the absolute number of cases averted in the US. The study by Clay et al. included assumptions around behavioral adaptation following the outbreak’s initiation among the GBMSM community in Washington, DC that were informed methods that had not been validated and may not be representative or generalizable to the wider US population [20]. The study by Zheng et al. was designed to project the case numbers for the US outbreak after 15 July 2022, which was while the epidemic curve was still on the rise. Unbeknownst to the researchers at that time, the outbreak would experience a downturn shortly after, with cases peaking on August 7th and declining quickly [22]. The model was found to greatly overestimate the counterfactual case estimate compared with case numbers reported by the CDC (2,392,548 vs. 30,093), presumably because the model did not account for the decline in US cases [7,22] (Table 2).

## 4. Discussion

This systematic review summarizes the current evidence from modeling studies of the public health impact of the mpox vaccine during the 2022 outbreak published in peer-reviewed journals from 2022 to July 2024. Specifically, we sought to determine how many cases the vaccine was estimated to have prevented during this outbreak, in conjunction with or independently of any behavioral changes made after individuals became aware of the mpox outbreak and their own risk. We analyzed nine studies which employed a variety of modeling methods to answer this question, or a related one. Direct comparisons between all nine models is challenging due to the heterogeneity of structure, objective, parameterization, epidemiological data used, and assumptions made. The estimates from the four papers that assessed the vaccine’s impact alone on the 2022 outbreak ranged from 9.8% to 79% of cases [17,18,19,20]. In some studies, it was clear why their estimate might have varied compared to another study’s estimate. There were variations in the assumed vaccine efficacy, the timing of the vaccination campaign, proxies used to assign risk status to populations, assumptions regarding population mixing, and behavior following mpox diagnosis. In other cases, the reason for large discrepancies between estimates is unclear, and likely lies within the model structure, transition probabilities, and parameterization.

Only one study (Shamier et al.) was identified which sought to estimate the effect the 2022 vaccination campaign would have on a potential future outbreak [21]. This is an important study as it aims to understand the broader long-term impact of immunity conferred by prior infections and vaccination among this population. The study found that in the absence of a vaccination campaign, there was only a marginal decrease in cumulative cases (7.1%); however, in a scenario with vaccination and no change to behavior, a future outbreak was projected to result in 74% fewer cases. Subject to differences in local vaccination strategies and patterns of behavioral changes, as well as differences in transmission dynamics, these findings may be applicable to other regions where future mpox outbreaks may occur, including current countries where outbreaks are ongoing. However, the assumptions regarding duration of protection reflect that of childhood smallpox vaccination against smallpox disease and must be updated as we continue to learn more about mpox and vaccine durability. At present, there is no clear immunological correlate of protection, with the available 1- and 2-dose immunogenicity data not predictive of the strong real-world vaccine effectiveness across dose schedules demonstrated in the 2022 outbreak [32]. As our understanding of mpox evolves, our modeled results must too.

Other modeling studies used more hypothetical approaches to estimate the impact of vaccination on mpox transmission and cumulative case numbers. They contained scenarios of varying vaccination coverage, optimal strategies included targeted and ring vaccination, and the impact of vaccination in combination with other countermeasures [22,23,24,25]. These modeling analyses offer key insights into the vaccine’s hypothetical impact but did not measure the impact of the vaccine during the 2022 outbreak explicitly.

Though mpox is continuing to spread in endemic countries, none of the included modeling studies estimated the impact of the vaccine in these countries. The population impact of the mpox vaccine is easiest to do in settings where high-quality, consistent epidemiologic surveillance data are available. When examining the landscape of published models estimating the impact of vaccination on mpox outbreaks across the globe, there was a notable dearth of models focused on populations in historically endemic areas. Countries on the African continent are experiencing significant ongoing outbreaks and sustained person-to-person transmission, particularly in the DRC [33]. The importance of understanding the vaccine’s potential impact for these ongoing public health emergencies is clear, but there is a gap due to a lack of context-specific modeling in endemic settings. The study by Savinkina et al. aimed to assess the impact of various potential vaccination strategies in the DRC specifically [34]. This is the first known modeling analysis of the outbreak in the DRC, making it an important study for our understanding of the impact of mitigation efforts globally. The authors explored the hypothetical impact of different vaccination approaches on the number of cases in the 2023 outbreak in the DRC [34].

Most recently, a modeling study explored vaccination strategies for achieving outbreak control in sub-Saharan Africa which incorporated DRC-specific demographics, contact patterns, and historical smallpox vaccination coverage to estimate the country-specific impact of a one-time mass vaccination campaign [35]. As context-specific estimates cannot be easily translated to other settings due to highly variable transmission dynamics, population demographics and mixing patterns, among other factors, country- or region-specific studies like these are needed to assess the impact of the mpox vaccine in countries where mpox is endemic. It is crucial that model structure, inputs and assumptions accurately reflect the specific context they are attempting to describe. This might include very different patterns of sexual transmission, as well as transmission in other contexts such as the household, medical settings and zoonotic exposures, in addition to different demographic features and sizes of high-risk populations [36,37,38]. Lack of robust surveillance and other data, as well as a paucity of models using data specific to the countries currently experiencing outbreaks of mpox, is a limitation in establishing an optimal vaccination strategy as part of the public health response in many endemic countries.

This SLR provides an overview of published models which estimate the impact of the vaccine to date. Various modeling approaches were used, and due to heterogeneity of populations, assumptions, and modeling methods, studies could not be quantitatively combined into a single estimate of the public health impact of the mpox vaccine. Despite this heterogeneity of estimates and methods, the review provides insight into the impact the mpox vaccine had during this previous global outbreak of mpox disease, as well as a solid understanding of the impact it may have on preparing for and responding to future outbreaks. One study yielded a realistic estimate for the proportion of cases averted in the US, using appropriate data sources and reasonable assumptions, which allows for approximation of the impact the vaccine had in the US during the 2022 mpox outbreak [17].

There are limitations with all modeling, and the assumptions and data utilized in each of the models included in this review have the potential to bias the results and predictions. A model may not be appropriate for extrapolation to other contexts due to population-specific demographics and past vaccination status, behavioral assumptions, and gaps in available surveillance data.

The first step in modeling an intervention is understanding how and why the disease is moving through the target population. Without this, a model cannot be reliably used to guide a vaccination strategy or estimate when an epidemic downturn will occur. Even the most sophisticated model cannot compensate for weak epidemiological inputs, thus limiting their utility where these data are limited or unavailable.

## 5. Conclusions

Mpox remains a global threat, highlighting the continued urgency to address and prevent further transmission and spread of the disease. No other systematic review has sought to consolidate the impact of the mpox vaccine on case numbers during the 2022 outbreak, or on possible future outbreaks. This review summarizes the current evidence on predicted case numbers in the absence of vaccination. Models vary greatly due to the model structure, underlying population context, and assumptions made.

Context-specific models are needed, as results cannot be easily translated from one setting to another. Consistency and reliability of models which investigate the public health impact of the mpox vaccine may improve as more is known about baseline immunity against mpox, risk factors, duration of protection, and transmission dynamics. Findings across all models demonstrate the importance of the combination of vaccination and public health interventions in mitigating current outbreaks of mpox and preventing future outbreaks.

## Figures and Tables

**Table 1:** Characteristics of Included Studies.

Study	Objective	Methods	Results	Key Parameters	Setting
Impact of the Vaccine During the 2022 Outbreak					
Lin, 2024 [17]	Proportion of cases averted by vaccination and risk behavior modification	Deterministic transmission compartmental model	Vaccines (2-dose campaign) could prevent 21.2% of cases (approximately 8096 cases); Behavior change: 15.4%; Both: 64.0% (approximately 53,499 cases)	R_0_ = 3.88 (high-risk) R_0_ = 0.39 (low-risk)	US
Zhang X, 2024 [18]	Estimate the effect of vaccination on the outbreak	Structured dynamic compartmental transmission model	Vaccination marginally increased the number of infections prevented (approx. 185 cases) but minimized a resurgence in cases from Jan 2023; could have averted 4x more if initiated earlier	R_0_ (homogenous) = 1.41–2.17 R_0_ (structured) = 1.94 (high-risk) and 0.67 (low-risk)	England
Brand, 2023 [19]	Make projections of future mpox incidence over a medium-term time horizon (26 weeks)	Bayesian, compartmental transmission model	Vaccination did not cause mpox incidence to turn over; however, a rebound in cases due to behavior reversion was prevented by high-risk group-targeted vaccination	R_0_ (GBMSM pop) = 5.16 (2.96–9.24) R_0_ (Overall) = 5.16 (2.96–9.24)	UK
Clay, 2024 [20]	Estimate the relative effects of behavioral adaptation and vaccination on the 2022 outbreak, and the theoretical impact if vaccines had been distributed earlier	Dynamic network transmission model incorporating both vaccine administration data and sexual partner acquisition	Initial declines in cases were likely caused by behavioral adaptations, but vaccination alone averted 79% (IQR: 64–88%) of cases, compared with behavioral adaptation alone (25% (IQR: 10–42%))	VE (first dose) = 75.2% VE (second) = 85.9%	US
Impact of the Vaccine on a Future Outbreak					
Shamier, 2024 [21]	Future outbreak scenarios based on seroprevalence data	Stochastic transmission compartmental model	Marginal decrease in cases due to vaccine: 1427 vs. 1321	Reduction in infection risk = 85% for historically vaccinated individuals (i.e., smallpox); 78% for recently vaccinated	Netherlands
Impact of a Hypothetical Vaccination Program or Strategies					
Zheng, 2022 [22]	Estimate the impact of diagnostic testing interventions and ring vaccination on cumulative cases	Epidemic dynamical model	Ring vaccination of 20% of exposed contacts reduces cumulative cases by 61.1% by end of 2022 If 40%, then 78.3% reduction; if 60%, then 81.8% reduction	Smallpox vaccine VE = 85%	US
Knight, 2022 [23]	Determine the optimal vaccination allocation strategy to minimize cumulative cases	Deterministic compartmental mpox virus transmission model	A limited mpox vaccine supply would avert more early infections when prioritized to larger networks with more initial infections or had a higher R_0_	Mpox vaccine VE = 85%	Canada
Gan, 2023 [24]	Simulate outbreaks and demonstrate value of pre-emptive vaccination before arrival of the virus	Individual-based SEIR compartmental model	Mass vaccination can reduce total cases by 22.3% to 96.1%. Targeted vaccination: cases can be reduced by 8.4% to 66.9% For mass vaccination the average number of cases averted per vaccine dose: 0.82 Singapore, 0.96 Hong Kong, and 0.85 Sydney	VE: 85% (Sensitivity analysis VE = 66%)	Singapore, Hong Kong, Sydney
Zhang L, 2024 [25]	Simulate global mpox transmission and countermeasure scenarios for the 2022 outbreak	Modified SEIR model	A 20% vaccination rate by the end of 2022 could have reduced mpox infection rates by 16%; a 30% rate could have reduced it by 29%	Mpox vaccine VE = 78%	Various

GBMSM—gay and bisexual men who have sex with men; IQR—interquartile range; R_0_—basic reproduction number; SEIR—susceptible, exposed, infected, recovered; UK—United Kingdom; US—United States; VE—vaccine efficacy.

**Table 2:** Summary of Cases Averted.

			Proportion of Mpox Cases Averted		
Objective	Study	Country or City	Due to Vaccine Alone	Due to Behavioral Changes Alone	Due to Behavioral Changes and Vaccination
Impact of the Vaccine During the 2022 Outbreak	Lin, 2024 [17]	US	21.2%	15.4%	64.0%
	Zhang X, 2024 [18]	England	9.8%	98%	98.1%
	Brand, 2023 [19]	UK	45–53% *	-	-
	Clay, 2024 [20]	Washington DC	79%	25%	84%
Impact on a Future Outbreak	Shamier, 2024 [21]	Netherlands	74%	-	-
Impact of a Hypothetical Vaccination Program or Strategies	Zheng, 2022 [22]	US	61.1–81.8% ^†^	-	-
	Knight, 2022 [23]	Toronto-like city Ontario-like city	Displayed in figure only	-	-
	Gan, 2023 [24]	Singapore Hong Kong Sydney	25–78% ** 29–96% ** 22–71% **	-	-
	Zhang L, 2024 [25]	Various	16–29% ^‡^	-	-

UK—United Kingdon; US—United States. * Return to normal behavior in 12 and 4 weeks. ^†^ 20–60% vaccination rate for contacts for a ring vaccination strategy. ** 20–30% vaccination rate. ^‡^ 25–80% vaccination rate.

## Data Availability

The original contributions presented in this study are included in the article/Supplementary Materials. Further inquiries can be directed to the corresponding author.

## Supplementary Materials

The following supporting information can be downloaded at: https://www.mdpi.com/article/10.3390/idr17050124/s1, Table S1: MEDLINE Search Strategy (implemented 10 January 2024).

## Abbreviations

The following abbreviations are used in this manuscript:

CDC	Centers for Disease Control and Prevention
CI	Confidence Interval
COVID-19	Coronavirus Disease 2019
DRC	Democratic Republic of the Congo
EUA	Emergency Use Authorization
EU	European Union
EUL	Emergency Use Listing
FDA	Food and Drug Administration
GBMSM	Gay and Bisexual Men who have Sex with Men
IQR	Interquartile Range
MPXV	Monkeypox Virus
MVA-BN	Modified Vaccinia Ankara-Bavarian Nordic
PHEIC	Public Health Emergency of International Concern
PI	Prediction Interval
PRISMA	Preferred Reporting Items for Systematic Reviews and Meta-Analyses
R_0_	Basic reproductive number
SEIR	Susceptible, Exposed, Infected, Recovered
SLR	Systematic Literature Review
UK	United Kingdom
US	United States
VE	Vaccine Efficacy
WHO	World Health Organization
