# Supplementary material for: Public Health Impact of the MVA-BN Vaccine During the 2022 Mpox Outbreak: A Systematic Review
Source: Infect Dis Rep. 2025 Oct 7;17(5):124. doi: 10.3390/idr17050124 (PMC12564786; doi:10.3390/idr17050124)
Supplement: Supplementary file 1 [file idr-17-00124-s001.zip › idr-3833576-supplementary.pdf]

Supplemental Table S1. MEDLINE Search Strategy (implemented 10 January 2024).

Search	Terms	Citations
#1: Disease	mpox[tiab] OR monkeypox[tiab]	4,839
#2: Intervention	#1 AND (JYNNEOS[tiab] OR vaccin*[tiab] OR immunization[tiab] OR immunize[tiab])	1,652
#3: Study Type Exclusions	#2 NOT ("case reports"[pt] OR "comment"[pt] OR "editorial"[pt] OR "review"[pt] OR "clinical trial protocol"[pt] OR "clinical trial")	1,156
#4: Non-Human Exclusions	#3 NOT ("Animals"[Mesh] NOT "Humans"[Mesh])	1,031
#5: Date Range	#7 AND Filters: from 2022 - 2024	801
**Search Filters**	**Filters: English**	**792**
